# Polyphenol Profiling, Antibacterial Activity, and In Vitro Cytotoxic Effects of a Diethyl Ether Fraction from Juniper Cone Berries

**DOI:** 10.3390/molecules31213910

**Published:** 2026-10-29

**Authors:** Alina Arabela Jojić, Diana Uţu, Gabriela Antal, Alexandra Mioc, Delia Muntean, Laurian Vlase, Ana-Maria Vlase, Elena-Alina Moacă, Raluca Vodă, Codruța Marinela Şoica, Diana-Simona Tchiakpe-Antal

**Affiliations:** 1 Department of Pharmaceutical Botany, Faculty of Pharmacy, “Victor Babes” University of Medicine and Pharmacy Timisoara, 2nd Eftimie Murgu Square, 300041 Timisoara, Romania; alina.jojic@umft.ro (A.A.J.); diana.antal@umft.ro (D.-S.T.-A.); 2 Department of Pharmacology-Pharmacotherapy, Faculty of Pharmacy, “Victor Babes” University of Medicine and Pharmacy Timisoara, 2nd Eftimie Murgu Square, 300041 Timisoara, Romania; alexandra.mioc@umft.ro (A.M.); codrutasoica@umft.ro (C.M.Ş.); 3 Research Centre for Experimental Pharmacology and Drug Design (X-Pharm Design), “Victor Babes” University of Medicine and Pharmacy Timisoara, 2nd Eftimie Murgu Square, 300041 Timisoara, Romania; 4 Discipline of Microbiology, Faculty of Medicine, “Victor Babes” University of Medicine and Pharmacy Timisoara, 2nd Eftimie Murgu Square, 300041 Timisoara, Romania; muntean.delia@umft.ro; 5 Discipline of Pharmaceutical Botany, Faculty of Pharmacy, “Iuliu Hațieganu” University of Medicine and Pharmacy, Victor Babeș Street, 400012 Cluj-Napoca, Romania; laurian.vlase@umfcluj.ro (L.V.); gheldiu.ana@umfcluj.ro (A.-M.V.); 6 Department of Toxicology and Drug Industry, Faculty of Pharmacy, “Victor Babes” University of Medicine and Pharmacy Timisoara, 2nd Eftimie Murgu Square, 300041 Timisoara, Romania; alina.moaca@umft.ro; 7 Faculty of Chemical Engineering, Biotechnologies and Environmental Protection, Politehnica University Timișoara, Vasile Pârvan No. 6, 300223 Timisoara, Romania; raluca.voda@upt.ro

**Keywords:** *Juniperus communis*, diethyl ether fraction, protocatechuic acid, melanoma, A375 cells, HaCaT keratinocytes, antibacterial effects

## Abstract

Modern research on medicinal plants aims to expand their applications beyond traditional usage. Juniper (*Juniperus communis* L.) cone berries have a background in the phytotherapy of skin diseases. The present study investigated a diethyl ether fraction (JC-DEE) obtained by sequential solvent partitioning of an ethanolic extract from *Juniperus communis* L. cone berries. Phytochemical characterization was performed using Fourier-transform infrared (FTIR) spectroscopy, HPLC–MS, and total phenolic content determination. The antimicrobial activity of JC-DEE and its effects on cell viability and morphology in HaCaT keratinocytes and A375 melanoma cells were evaluated, alongside an exploratory DPPH spectrophotometric assessment. Targeted HPLC–MS analysis identified eleven selected constituents, seven of which were quantified within the validated calibration range, with apigenin (1.971 ± 0.150 µg/mL), protocatechuic acid (1.070 ± 0.049 µg/mL), and luteolin (0.560 ± 0.046 µg/mL) showing the highest concentrations in the analyzed solution. The antimicrobial assay revealed limited inhibitory effects against the tested microorganisms, with inhibition zones ranging from 7 to 10 mm. Exploratory DPPH measurements revealed time- and concentration-associated variations in apparent absorbance reduction. In the cellular assays, JC-DEE produced a concentration-dependent reduction in viability in both human A375 melanoma cells and HaCaT keratinocytes, with estimated IC_50_ values of 102.7 μg/mL and 129.3 μg/mL, respectively. Phase-contrast microscopy revealed concentration-related morphological alterations, while immunofluorescence analysis at 100 μg/mL demonstrated cytoskeletal and nuclear changes, including nuclear condensation and fragmentation, with apoptotic body-like structures particularly evident in A375 cells. Overall, JC-DEE represents a phytochemically characterized fraction exhibiting limited antimicrobial effects and concentration-dependent cytotoxicity in the investigated cellular models, while its antioxidant potential remains to be validated. Further studies are required to identify the constituents responsible for these effects, clarify the mechanisms of cell death, and assess selectivity and safety in additional non-malignant cellular models.

## 1. Introduction

*Juniperus communis* L. (*Cupressaceae*), commonly known as juniper, has a long history of use in traditional medicine, with its cone berries and other plant parts being employed for their antiseptic [1], anti-inflammatory [2], antioxidant [3], diuretic, and digestive properties [4]. Traditional applications also include the treatment of skin affections, supporting the ethnomedicinal relevance of juniper in dermatological conditions [5]. In Romanian traditional and historical medicine, *J. communis* has been used for a variety of medicinal purposes. Older Romanian medicinal literature records the use of juniper leaves (*Juniperi folia*) as a purgative and diuretic, while the wood (*Juniperi lignum*) was employed as a sudorific and was historically recommended for tuberculosis and gout. The pseudo-fruits (*Juniperi galbulus*) represented the most important medicinal part and were used in preparations intended for urinary, digestive, respiratory, and rheumatic complaints. Traditional preparations included alcoholic tinctures of the pseudo-fruits, while *Juniperi galbulus* was also incorporated into antirheumatic herbal teas. These historical uses illustrate the longstanding importance of *J. communis* in Romanian medicinal practice [6]. In addition to these traditional uses, experimental evidence has demonstrated the antimicrobial potential of *J. communis*, with both essential oils and non-volatile extracts exhibiting antibacterial activity against a range of pathogenic microorganisms. These effects have been associated with its chemically diverse bioactive constituents, particularly volatile terpenoids and phenolic compounds, although antimicrobial activity varies according to the plant material, extract composition, and tested microorganism [7]. Beyond its traditional applications, the broad phytochemical and pharmacological profile of *J. communis* provides a rationale for extending the investigation of this species toward other biomedical applications, including cancer research.

Cutaneous melanoma is one of the most aggressive forms of skin cancer, being characterized by a high metastatic potential and a substantial risk of mortality in advanced stages [8]. Despite major advances in melanoma management, including immune checkpoint inhibitors and molecularly targeted therapies, therapeutic resistance, disease recurrence, and treatment-associated toxicity remain important clinical challenges [9]. These limitations continue to stimulate the search for complementary therapeutic strategies and novel bioactive compounds capable of interfering with multiple mechanisms involved in melanoma progression. In this context, natural products have attracted increasing attention as potential sources of structurally diverse compounds with antiproliferative, pro-apoptotic, antioxidant, anti-inflammatory, and anti-metastatic properties [10]. Recent reviews have emphasized the considerable preclinical evidence supporting the potential of plant-derived products and phytochemicals in melanoma research, while also highlighting the need for further studies linking phytochemical composition to biological activity [11].

Plant-derived natural products have attracted considerable interest in cancer chemoprevention because they provide structurally diverse bioactive compounds capable of acting on cellular and molecular processes associated with carcinogenesis. Phytochemicals, including polyphenols, flavonoids and terpenoid compounds, have been investigated for their ability to modulate oxidative stress, inflammation, apoptosis, cell-cycle regulation, angiogenesis, and signaling pathways involved in tumor growth and progression [12,13]. In melanoma research, several classes of phytochemicals, including phenolic compounds, flavonoids, terpenoids, carotenoids, and constituents of essential oils, have demonstrated their antiproliferative and cytotoxic effects in established tumor cell lines [14,15]. Nevertheless, much of the available evidence remains preclinical, emphasizing the need for further mechanistic and translational investigations before their potential role in melanoma prevention or therapy can be established [15]. The role of oxidative stress in melanoma is complex and stage-dependent: increased ROS may contribute to tumor initiation, whereas established melanoma cells can develop adaptive antioxidant mechanisms that support their survival under oxidative conditions. Consequently, antioxidant compounds may be particularly relevant in a chemopreventive context, while pro-oxidant strategies have been proposed as potentially more appropriate for targeting established melanoma. Thus, antioxidant activity should be regarded as one component of a broader chemopreventive profile rather than as direct evidence of anticancer efficacy [16].

The anticancer potential of *J. communis* has been investigated using both volatile and non-volatile preparations in several experimental tumor models. Cone berry essential oil produced a concentration-dependent reduction in cell viability and long-term clonogenic survival in HeLa cervical cancer and HCT 116 colorectal cancer cells, while mechanistic analyses indicated activation of the intrinsic apoptotic pathway; additional effects included S-phase arrest in HeLa cells and inhibition of survival-related signaling in HCT 116 cells [17]. Non-volatile preparations have also shown anticancer activity: an aqueous extract obtained from *J. communis* berries decreased cell viability in A549 lung cancer, 22RV1 and DU145 prostate cancer, and HepG2 hepatocellular carcinoma cells, concomitantly with activation of p53 signaling and inhibition of the PI3K/Akt pathway [18]. In colorectal cancer models, *J. communis* extract reduced the viability of human HT-29 and murine CT-26 cells and induced G0/G1 cell-cycle arrest and apoptosis; importantly, its antitumor effect was also demonstrated in vivo, where treatment suppressed tumor growth in a CT-26 tumor-bearing mouse model through reduced proliferation and increased apoptosis [19]. Gao and co-workers were the first to report that a Juniper (plant part not detailed) extract obtained by steam distillation suppressed melanoma tumorigenesis in vitro and in vivo, reducing tumor burden through mechanisms involving cell-cycle regulation and induction of apoptosis. The tested essential oil, having as main constituents alfa-pinene, citronellyl acetate, limonene and terpinolene, also affected proteins associated with angiogenesis and metastasis, while relatively low systemic toxicity was reported in the experimental models [20]. These findings indicate that *J. communis* represents a relevant natural source for investigating anti-melanoma activity. However, juniper contains a vast array of non-volatile components as well [21]. Such complex mixtures of constituents require prior fractionation of their compounds in order to study the biological effects in a meaningful way. Various solvents extract compounds according to their polarity, yielding fractions with distinct phytochemical profiles and biological activities. In our previous research, we demonstrated that the water-soluble fraction of *J. communis* pseudo-fruits had a significant antiproliferative effect on A375 melanoma cells in the MTT assay [22,23].

The findings of Gao et al. [20] and our own previous research provided a rationale for further exploring the anti-melanoma potential of *J. communis* cone berries. The present investigation extends previous fraction-oriented research [22,23] by focusing on the diethyl ether fraction (JC-DEE), obtained through the same extraction and fractionation procedure but not characterized in the previous publications. This enables a comparison of fractions with different polarities and provides additional insights into their relative composition and biological properties. The physicochemical properties of extraction solvents influence the distribution of plant secondary metabolites among different fractions [24,25,26]. Diethyl ether is a relatively low-polarity solvent; the resulting fraction may contain constituents with different physicochemical properties, including phenolic acids, flavonoid aglycones, and other extractable metabolites. Accordingly, this study aimed to characterize the phytochemical profile of JC-DEE, investigate its antimicrobial activity and effects on the metabolic activity and morphology of A375 human melanoma cells, and document preliminary DPPH spectrophotometric observations without inferring validated antioxidant activity. Ha-CaT immortalized human keratinocytes were included as a non-malignant comparator. Accordingly, the present investigation focused on the diethyl ether fraction obtained.

## 2. Materials and Methods

### 2.1. Chemicals and Reagents

Absolute ethanol (99.5%; Riedel-de Haën, Fisher Scientific, Vantaa, Finland) was used to prepare the crude ethanolic extract of *Juniperus communis* L. cone berries. Petroleum ether and diethyl ether, used for sequential liquid–liquid partitioning, were purchased from Sigma-Aldrich (St. Louis, MO, USA). For total phenolic content determination, chlorogenic acid (99%; Sigma-Aldrich, St. Louis, MO, USA) was used as the calibration standard, sodium carbonate (Na_2_CO_3_, 99%) was obtained from Carl Roth (Dautphetal, Germany), and Folin–Ciocâlteu reagent was purchased from Merck (Darmstadt, Germany).

Antioxidant activity was evaluated using 2,2-diphenyl-1-picrylhydrazyl (DPPH; Sigma-Aldrich, Steinheim, Germany), with ascorbic acid (Lach-Ner, Prague, Czech Republic) employed as the reference antioxidant. All chemicals used in these procedures were of analytical grade.

For quantitative analysis by liquid chromatography–mass spectrometry (LC/MS), 82 phenolic compound reference standards were commercially available from Sigma Aldrich Chemie GmbH (Schnelldorf, Germany), Carl Roth GmbH (Karlsruhe, Germany), Merck (Darmstadt, Germany), Supelco (Bellefonte, PA, USA), and Extrasynthese (Genay, France). MS-grade methanol and MS-grade acetic acid were obtained from Merck KGaA (Darmstadt, Germany). Ultrapure water was obtained using a Milli-Q Direct 8 water purification system (Millipore, Bedford, MA, USA).

### 2.2. Plant Material and Preparation of the Diethyl Ether Fraction

Mature dried pseudo-fruits of *Juniperus communis* L. were purchased from STEFMAR PRODUCTIE S.R.L. (Râmnicu Vâlcea, Romania) as a commercially packaged herbal product (50 g per package; product barcode: 6422579000788). Botanical authentication was performed at the Department of Pharmaceutical Botany, Faculty of Pharmacy, “Victor Babeș” University of Medicine and Pharmacy Timișoara, Romania, and a voucher specimen was deposited in the institutional herbarium (JAA-01-23).

A total of 300 g of dried pseudo-fruits was mechanically comminuted using an IKA A11 analytical grinder (IKA, Staufen, Germany). The powdered plant material was immersed in 1 L of absolute ethanol and maintained for 24 h at room temperature (22 ± 2 °C). The extraction was subsequently assisted by ultrasonication for 20 min using an Elmasonic S120 ultrasonic bath (Elma Schmidbauer GmbH, Singen, Germany). Following sonication, the liquid phase was separated from the plant residue by filtration through Whatman Grade 4 filter paper. The solvent was removed under reduced pressure at 35 °C using a Laborota 4000eco rotary evaporator (Heidolph Instruments GmbH & Co. KG, Schwabach, Germany). The residual plant material underwent two additional extraction cycles under identical conditions, using a further 2 L of ethanol. The filtrates obtained from the three extraction cycles were pooled and concentrated to dryness, providing 56.69 g of crude ethanolic extract.

For solvent partitioning, 20 g of the crude extract were dispersed in 100 mL of distilled water and transferred to a separatory funnel. The resulting aqueous suspension was initially extracted three times with petroleum ether (150 mL per extraction), resulting in highly lipophilic constituents. The petroleum ether layers were combined and concentrated under reduced pressure to obtain 8.61 g of the petroleum ether fraction (JC-PE), corresponding to a yield of 43.05% (*w*/*w*) relative to the crude extract subjected to partitioning. The remaining aqueous phase was subsequently extracted three times with diethyl ether (150 mL per extraction). The combined diethyl ether layers were concentrated under reduced pressure until complete solvent removal, yielding 3.52 g of the diethyl ether fraction, equivalent to 17.60% (*w*/*w*) of the crude extract. When calculated relative to the initial dried plant material, the corresponding yields were 8.13% and 3.33% (*w*/*w*) for JC-PE and JC-DEE, respectively.

### 2.3. FTIR Spectroscopic Characterization

**Figure 1:**
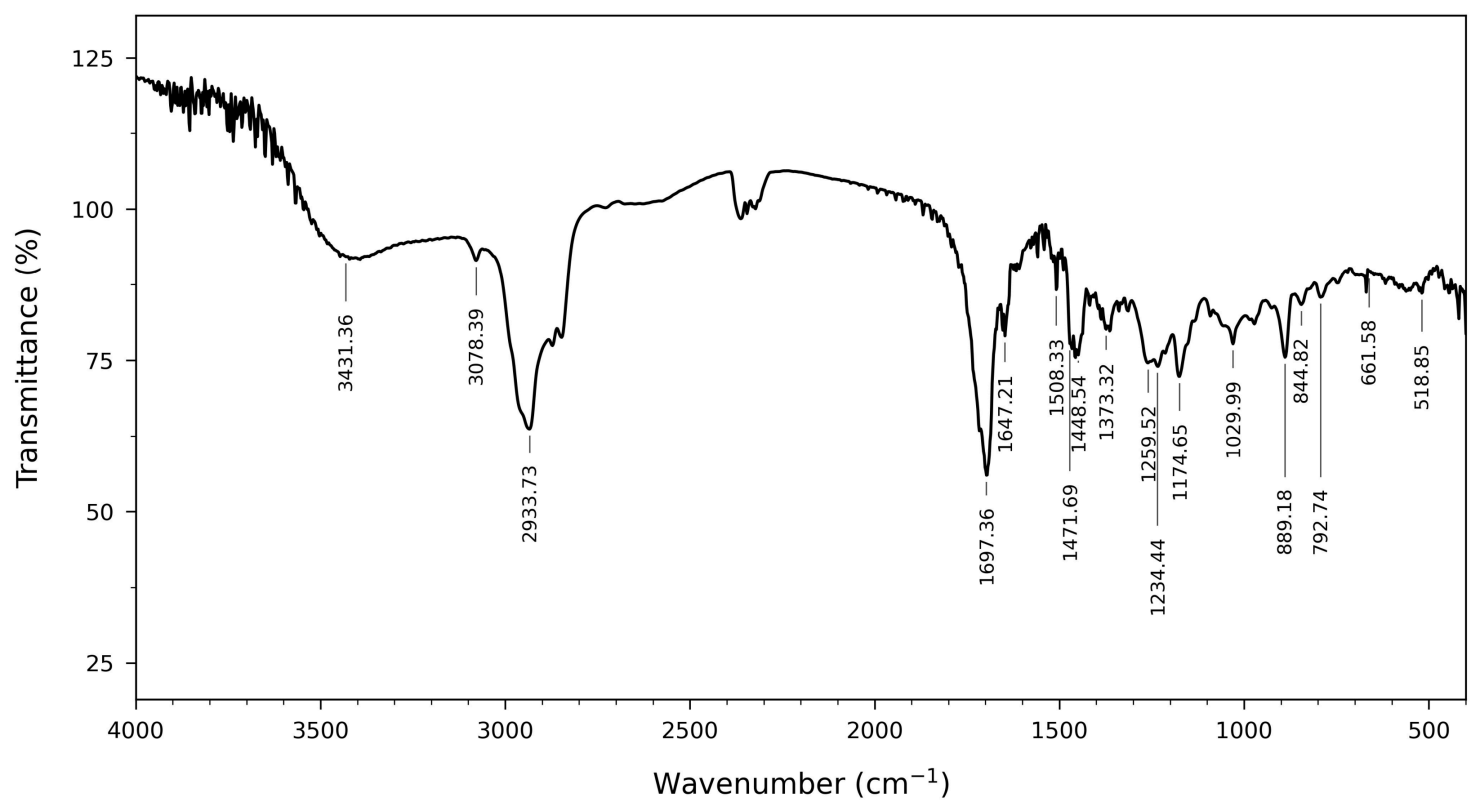
FTIR transmittance spectrum of JC-DEE replotted from the exported data. Labels reproduce the 18 positions on the original instrument plot; downward %T minima represent absorption maxima. The plotted data were not smoothed or baseline-corrected.

The dried *Juniperus communis* diethyl ether fraction was analyzed by Fourier-transform infrared (FTIR) spectroscopy using an IRPrestige-21 spectrometer (Shimadzu, Kyoto, Japan). The fraction was incorporated into KBr pellets and measured at room temperature over 4000–400 cm^−1^ at a nominal resolution of 4 cm^−1^. The exported data were expressed as percentage transmittance (%T). The spectrum was replotted without smoothing or baseline correction; the band positions shown in Figure 1 were transcribed from the original instrument-annotated spectrum. Vibrational assignments were treated as tentative because bands from constituents of a plant fraction can overlap [27].

### 2.4. HPLC-MS Analysis of Phenolic Constituents

Chromatographic analyses were performed using a Shimadzu Nexera X3 HPLC system (Shimadzu Corporation, Kyoto, Japan) equipped with two LC-40D X3 pumps with integrated degassing, a SIL-40C X3 autosampler, and a CTO-40S column oven. The LC system was coupled to a Shimadzu LCMS-9050 quadrupole time-of-flight mass spectrometer. Data acquisition, processing, spectral-library matching, and quantitative evaluation were performed using LabSolutions software, version 5.137 (Shimadzu Corporation).

The analytical procedure was adapted from previously published methods for the LC-MS analysis of phenolic compounds [28,29,30]. For HPLC–MS analysis, 3 mg of dried JC-DEE fraction was solubilized in 1 mL methanol and the samples thus prepared were analyzed without further dilution. Chromatographic separation was achieved on a Zorbax SB-C18 column (100 mm × 3.0 mm i.d., 3.5 µm; Agilent Technologies) maintained at 48 °C. Mobile phase A consisted of 0.2% (*v*/*v*) acetic acid in water, and mobile phase B consisted of 0.2% (*v*/*v*) acetic acid in methanol. The gradient increased linearly from 5% to 70% B over 0–30 min, then maintained at 70% B until 32 min, followed by a 4-min re-equilibration at 5% B. The flow rate was 1.0 mL/min and the injection volume was 1 µL.

The LCMS-9050 was operated with electrospray ionization in negative-ion mode. The interface voltage was −2.0 kV; the interface, desolvation-line, and heat-block temperatures were 300, 300, and 400 °C, respectively. Nebulizing-gas flow was 3 L/min, while heating-gas and drying-gas flows were each 12 L/min. Air was used as heating gas and nitrogen as nebulizing and drying gas.

Multiple scheduled acquisition events were used. A continuous high-resolution full-scan MS event was acquired from 0.8 to 31.0 min and was accompanied by data-dependent MS/MS acquisition (DDA) over the same chromatographic interval. DDA spectra were used for structural annotation or confirmation. For analytes with authentic standards, a compound-specific scheduled quantitation event was acquired from 0.8 min before to 0.8 min after the standard retention time, using HR-MS or HR-MS/MS (Table 1, data shown for identified compounds only). A ±10 ppm extraction window was used during initial ion searching and data processing. To maintain high mass accuracy throughout each run, an internal calibration event was acquired between 0.1 and 0.3 min, before the chromatographic dead time, using a 400 mg/L sodium iodide calibration solution (P/N 220-91239-90; Shimadzu Corporation). Mass correction was applied automatically to the acquired spectra by LabSolutions.

For the phenolic compounds, external calibration based on authentic standards was performed over 0.05–50 µg/mL. The validated limit of quantification (LOQ) was 0.050 µg/mL for every analyte, corresponding to the lowest calibration standard. Peak area was used as the analytical response, and calibration data were fitted by linear regression; the corresponding slopes, intercepts, and correlation coefficients are reported in Table 2 (data shown for identified compounds only). Method validation included linearity, sensitivity, precision, and accuracy. Operational limits of detection were defined as the lowest tested concentration providing signal-to-noise ratios of at least 3.

### 2.5. Determination of Total Phenolic Content

The total phenolic content (TPC) of JC-DEE was determined using the Folin-Ciocâlteu colorimetric assay according to Conforti et al. [31]. JC-DEE was prepared at a concentration of 2 mg/mL in a mixture of acetone, methanol, distilled water, and acetic acid (40:40:20:0.1, *v*/*v*/*v*/*v*). An aliquot of 200 μL of the sample solution was mixed with 1 mL of Folin-Ciocâlteu reagent and 1 mL of 7.5% (*w*/*v*) Na_2_CO_3_ solution. The reaction mixture was maintained for 2 h, after which the absorbance was recorded at 726 nm using an Agilent BioTek Synergy H1 Hybrid Multi-Mode Reader (Agilent Technologies, Inc., Santa Clara, CA, USA).

Chlorogenic acid was used as the calibration standard, and the total phenolic content was expressed as milligrams of chlorogenic acid equivalents per gram of dried fraction (mg CAE/g JC-DEE). Three independent determinations were performed, and the results were reported as mean ± standard deviation (SD).

### 2.6. Exploratory DPPH Spectrophotometric Assessment

The JC-DEE fraction was subjected to an exploratory spectrophotometric assessment using the 2,2-diphenyl-1-picrylhydrazyl (DPPH) assay, following an experimental approach previously applied to *Juniperus communis* cone berries and other plant-derived extracts [22,32]. A 0.1 mM DPPH solution (Sigma-Aldrich, Steinheim, Germany) was prepared in 95% (*v*/*v*) ethanol and maintained protected from light at 4 °C until use. JC-DEE sample solutions were prepared at concentrations of 0.1, 0.3, 0.5, 0.8, and 1.0 mg/mL. These concentrations refer to the sample solutions before their addition to the DPPH reagent. Following the mixing of 0.3 mL of each sample solution with 2.7 mL of DPPH solution, the final JC-DEE concentrations in the 3.0 mL reaction mixture were 0.01, 0.03, 0.05, 0.08, and 0.10 mg/mL, respectively. Ascorbic acid (Lach-Ner, Prague, Czech Republic) was used as the reference antioxidant at an initial concentration of 0.1 mM (0.0176 mg/mL), corresponding to a final concentration of 0.01 mM in the reaction mixture.

For each determination, 0.3 mL of the corresponding JC-DEE solution was mixed with 2.7 mL of the ethanolic DPPH solution in a quartz cuvette. The change in absorbance was monitored at 517 nm using a UviLine 9400 spectrophotometer (SI Analytics, Mainz, Germany), with readings acquired at 5 s intervals over a total reaction period of 1200 s. A DPPH solution measured under the same experimental conditions, in the absence of JC-DEE or ascorbic acid, served as the reference control. The DPPH reference control consisted of 2.7 mL of the ethanolic DPPH solution, without the addition of sample solution or supplementary solvent. Thus, the control was not volume- or solvent-matched to the sample-containing reaction mixtures. The control absorbance was recorded over the same reaction period and used as the corresponding reference at each measurement time point. Extract-only blanks without DPPH were not included in the original experimental protocol; therefore, no independent correction for the intrinsic absorbance of JC-DEE at 517 nm could be applied. Furthermore, the difference in final DPPH concentration between the reference control (0.1 mM) and sample-containing mixtures (0.09 mM) introduced a dilution-related contribution to the measured absorbance differences. Because these sources of analytical interference could not be resolved, the measurements were retained exclusively as unvalidated preliminary spectrophotometric observations.

For descriptive purposes, the recorded spectrophotometric responses were expressed as an apparent DPPH absorbance reduction index (AR), calculated relative to the original unmatched reference control according to the following equation:

AR (%) = [(A_0_ − A_s_)/A_0_] × 100

where A_0_ represents the absorbance of the unmatched DPPH reference control at the corresponding measurement time point, and A_s_ represents the absorbance of the reaction mixture containing JC-DEE or ascorbic acid at the same time point. The calculated AR values represent unvalidated apparent absorbance differences rather than validated radical-scavenging percentages, as they were not corrected for the dilution-related difference in DPPH concentration or for sample-specific background absorbance. Accordingly, these indices cannot be used to quantify antioxidant potency or establish a validated concentration–response relationship. Values recorded at 5 s and 1200 s were retained to provide a descriptive comparison of the initial and final spectrophotometric observations.

### 2.7. Antimicrobial Activity

The antimicrobial activity of the JC-DEE fraction was evaluated against five reference microorganisms: *Staphylococcus aureus* ATCC 25923, *Streptococcus pyogenes* ATCC 19615, *Escherichia coli* ATCC 25922, *Pseudomonas aeruginosa* ATCC 27853, and *Candida parapsilosis* ATCC 22019 (Thermo Scientific, Waltham, MA, USA). The reference microorganisms were selected to include clinically relevant representatives of Gram-positive bacteria (*Staphylococcus aureus* and *Streptococcus pyogenes*), Gram-negative bacteria (*Escherichia coli* and *Pseudomonas aeruginosa*), and yeasts (*Candida parapsilosis*). This panel was intended to provide a preliminary assessment of the antimicrobial activity of JC-DEE against microorganisms with different cellular characteristics and susceptibility profiles, rather than a comprehensive evaluation of its antimicrobial spectrum. The experimental procedure was based on methodological principles derived from EUCAST and CLSI recommendations and adapted from previously established antimicrobial screening protocols [22,33,34].

Bacterial strains were cultured on Columbia agar supplemented with 5% sheep blood, whereas *C. parapsilosis* was maintained on Sabouraud agar containing chloramphenicol (Oxoid, Wesel, Germany). Microbial suspensions were prepared in sterile 0.85% NaCl solution and adjusted to a turbidity equivalent to a 0.5 McFarland standard, corresponding to approximately 1–2 × 10^8^ CFU/mL.

Antimicrobial screening was performed using the disk diffusion method, following a previously applied experimental approach [22,35]. Mueller–Hinton (MH) agar was used for bacterial testing, except for *S. pyogenes*, for which Mueller–Hinton agar supplemented with blood and β-NAD (MHF; Oxoid, Wesel, Germany) was employed. After inoculation of the agar surface with the corresponding microbial suspension, sterile 6 mm paper disks (BioMaxima, Lublin, Poland) were placed onto the plates and loaded with 5 μL of JC-DEE solution at a concentration of 20 mg/mL (corresponding to 100 μg of JC-DEE per disk). Gentamicin disks (120 µg/disk for *S. pyogenes* and 10 µg/disk for *S. aureus*, *E. coli*, and *P. aeruginosa*) were used as positive controls for antibacterial testing, whereas fluconazole disks (25 µg/disk) were employed as the positive control for antifungal testing against *C. parapsilosis*. The negative control consisted of sterile blank disks impregnated with an ethanol–water mixture (1:1, *v*/*v*). The plates were incubated at 35 °C for 24 h, after which antimicrobial activity was evaluated by measuring the diameter of the inhibition zones, including the diameter of the 6 mm paper disks. All antimicrobial activity determinations were performed in triplicate.

For the purposes of antimicrobial screening, an inhibition-zone diameter > 15 mm was predefined as the criterion for subsequent minimum inhibitory concentration (MIC) determination, in accordance with the previously applied experimental protocol [22,35]. This threshold was used solely as an experimental screening criterion and was not interpreted as a clinical susceptibility breakpoint.

### 2.8. Cell-Based Assays

#### 2.8.1. Cell Lines and Culture Conditions

Two human cell lines were employed for the in vitro evaluation of JC-DEE: immortalized human keratinocytes HaCaT (catalogue no. 300493; CLS Cell Lines Service GmbH, Eppelheim, Germany) and A375 human malignant melanoma cells (ATCC^®^ CRL-1619^TM^; American Type Culture Collection, Manassas, VA, USA).

Cells were routinely propagated in Dulbecco’s Modified Eagle Medium (DMEM) containing 10% fetal bovine serum (FBS) and 1% penicillin–streptomycin solution (10,000 U/mL). Cultures were maintained at 37 °C in a humidified incubator with an atmosphere of 5% CO_2_.

HaCaT cells were included as a non-malignant keratinocyte model for comparison with the A375 melanoma cell line.

#### 2.8.2. Assessment of Cell Viability by Alamar Blue Assay

The influence of the JC-DEE on cellular viability was assessed in HaCaT and A375 cells using the Alamar Blue assay. Cells were seeded in 96-well plates at 1 × 10^4^ cells/well and allowed to attach before treatment. JC-DEE was dissolved in dimethyl sulfoxide (DMSO) and subsequently diluted in culture medium to obtain final concentrations of 25, 50, 75, 100, 200, and 300 μg/mL. The final DMSO concentration was maintained at 0.5% (*v*/*v*) in all JC-DEE-treated wells. The corresponding vehicle control contained the same concentration of DMSO (0.5%, *v*/*v*) without JC-DEE. Under these experimental conditions, the vehicle did not measurably affect cell viability.

To assess potential interference with the assay reagents, cell-free controls containing culture medium with Alamar Blue reagent, culture medium with JC-DEE, and control wells with culture medium together with both JC-DEE and Alamar Blue reagent were evaluated under the same experimental conditions. JC-DEE did not induce any detectable changes in fluorescence. After 24 h of exposure, the JC-DEE-containing medium was removed and replaced with fresh culture medium before the addition of 10 μL of Alamar Blue solution (0.015%, *w*/*v*) to each well. Cells were then incubated for a further 3 h to allow conversion of the viability reagent. Fluorescence intensity was measured using a Synergy HTX Multi-Mode Reader (BioTek Instruments, Winooski, VT, USA) at an excitation wavelength of 528 nm and an emission wavelength of 590 nm.

Cell viability was expressed relative to the corresponding vehicle control (0.5% DMSO). All experiments were performed independently three times (*n* = 3 biological replicates), each biological replicate was assessed in three technical replicate wells/experiment. The technical replicate measurements were averaged and the resulting mean biological replicate was used for the statistical analysis.

#### 2.8.3. Immunofluorescence Analysis of Nuclear and Cytoskeletal Morphology

The effect of JC-DEE on cellular morphology, specifically on nuclear architecture and actin cytoskeletal organization, was further examined by immunofluorescence microscopy. HaCaT and A375 cells were exposed to 100 μg/mL JC-DEE for 24 h. Prior to the immunofluorescence staining protocol, the JC-DEE-containing medium was removed and the wells were washed with PBS. Staurosporine was dissolved in DMSO (500 µM stock solution) and was applied at a final concentration of 2.5 μM for 24 h, as a positive control for apoptosis induction. The final DMSO concentration in the staurosporine-treated samples was 0.5% (*v*/*v*); at this concentration, DMSO did not significantly affect cell morphology, compared to vehicle-free control wells. After the treatment period, the cells were fixed with 4% paraformaldehyde for 15 min and subsequently permeabilized with 0.1% Triton X-100 for 15 min. Nonspecific antibody interactions were minimized by blocking the samples with 3% bovine serum albumin (BSA) for 30 min at room temperature. The actin cytoskeleton was labelled with a mouse monoclonal anti-β-actin primary antibody (Product No. MA1-140; Thermo Fisher Scientific, Inc., Waltham, MA, USA) at a 1:2000 dilution. Fluorescent detection was subsequently achieved using an Alexa Fluor Plus 488-conjugated goat anti-mouse IgG (H+L) secondary antibody (Product No. A32723; Thermo Fisher Scientific, Inc.) diluted 1:500. Incubation with the secondary antibody was performed for at least 30 min under light-protected conditions. Nuclei were counterstained with Hoechst 33342 (Product No. 62249; Thermo Fisher Scientific, Inc.). Fluorescence images were acquired using an EVOS^TM^; M5000 Imaging System (Thermo Fisher Scientific) equipped with a 40× objective. Images were presented as individual fluorescence channels for β-actin (green) and Hoechst (blue), together with merged images.

#### 2.8.4. Statistical Analysis

Statistical analyses were conducted using GraphPad Prism version 5.5.0 (GraphPad Software, San Diego, CA, USA). Differences between JC-DEE-treated groups and the corresponding vehicle control were evaluated by one-way analysis of variance (ANOVA), followed by Dunnett’s multiple-comparison test. The technical replicates (*n* = 3) were averaged within each independent experiment, and only the resulting three biological replicate values (*n* = 3) were used for statistical analysis. Given the limited number of independent experimental replicates, formal tests of normality were not considered sufficiently informative. The suitability of the parametric approach was therefore assessed in relation to the experimental design, together with inspection of the distribution and variability of the data obtained. IC_50_ values were estimated by nonlinear regression using a logistic concentration–response model with variable slope and fixed asymptotes (top = 100%; bottom = 0%) with the program GraphPad Prism version 5.5.0 (GraphPad Software, San Diego, CA, USA). The model was fitted to log_10_-transformed JC-DEE concentrations. The data was normalized to the vehicle control, defined as 100%. However, the vehicle control (0 μg/mL) cannot be log-transformed; hence it was used for normalization but excluded from the regression. The IC_50_ estimates were reported with their corresponding 95% confidence intervals. Statistical significance was established at *p* < 0.05. Differences relative to the corresponding vehicle control were denoted as follows: *p* < 0.05 (*), *p* < 0.01 (**), and *p* < 0.001 (***).

## 3. Results

### 3.1. HPLC-MS Analysis of Polyphenolic Phytochemicals

HPLC–MS analysis of JC-DEE identified eleven targeted compounds, including hydroxybenzoic and hydroxycinnamic acids, flavonoid glycosides and aglycones, and the coumarin umbelliferone. Seven compounds were quantified within the validated calibration range; four were detected below the LOQ of 0.050 µg/mL. Compound identification was supported by chromatographic retention times and compound-specific MS precursor ions. Among the quantified constituents, apigenin exhibited the highest concentration (1.971 ± 0.150 µg/mL), followed by protocatechuic acid (1.070 ± 0.049 µg/mL) and luteolin (0.560 ± 0.046 µg/mL). Apigenin-7-glucoside (0.266 ± 0.023 µg/mL), vanillic acid, p-coumaric acid, and umbelliferone were also quantified. Gallic acid, ferulic acid, kaempferol-3-O-glucoside, and acacetin were detected below LOQ and are reported qualitatively in Table 3. Umbelliferone and acacetin were identified as minor constituents. Overall, the targeted HPLC–MS profile showed that JC-DEE was predominantly characterized by apigenin, protocatechuic acid, and luteolin, accompanied by lower amounts of structurally diverse phenolic compounds.

### 3.2. Total Phenolic Content

The total phenolic content of the JC-DEE fraction, determined using the Folin–Ciocalteu assay, was 114.22 ± 5.35 mg chlorogenic acid equivalents (CAE)/g extract.

When considered in relation to previously investigated fractions of the same *J. communis* extract, JC-DEE showed an intermediate phenolic content. Its TPC was slightly higher than that previously determined for the *n*-butanol fraction (104.73 ± 10.03 mg CAE/g extract) and higher than that reported for the aqueous fraction (70.75 ± 8.80 mg CAE/g extract), but markedly lower than that of the ethyl acetate fraction (361.08 ± 17.72 mg CAE/g extract) [22,23]. Thus, although the strongest phenolic enrichment occurred in the ethyl acetate fraction, the present findings demonstrate that a relevant proportion of Folin–Ciocalteu-reactive constituents also partitioned into the diethyl ether phase.

### 3.3. FTIR Spectroscopy

The spectrum of the JC-DEE fraction showed strong absorption maxima at 2933.73 and 1697.36 cm^−1^, consistent with aliphatic C–H stretching and carbonyl stretching, respectively (Figure 1; Table 4). A broad feature at 3431.36 cm^−1^ was compatible with O–H stretching, although moisture in KBr may contribute. The signal at 3078.39 cm^−1^ may arise from sp2 C–H groups in alkenic and/or aromatic structures. The 1647.21 cm^−1^ band can include alkene C=C stretching, with possible overlap from absorbed water; the band at 1508.33 cm^−1^ is compatible with aromatic ring vibrations.

Bands at 1471.69, 1448.54, and 1373.32 cm^−1^ are provisionally associated with C–H deformation modes. Signals at 1259.52, 1234.44, 1174.65, and 1029.99 cm^−1^ lie in an overlapping C–O stretching region. The absorptions at 889.18, 844.82, and 792.74 cm^−1^ may include out-of-plane C–H vibrations of alkenic and/or aromatic groups. The feature labelled at 661.58 cm^−1^ requires caution because atmospheric CO2 absorbs in this region; the weak 518.85 cm^−1^ feature is left unassigned. These positions describe functional-group possibilities and do not identify individual compounds. Overall, the FTIR profile supports the presence of hydroxylated, carbonyl-containing, and aromatic constituents, in agreement with the phenolic compounds identified by HPLC–MS.

### 3.4. Exploratory DPPH Spectrophotometric Observations

The DPPH assay provided preliminary spectrophotometric observations regarding the behavior of JC-DEE-containing reaction mixtures over a 1200 s monitoring period. Given the methodological limitations associated with the unmatched reference control and the absence of extract-specific blanks, the calculated responses were retained exclusively as apparent absorbance reduction indices rather than validated measurements of radical-scavenging activity.

**Figure 2:**
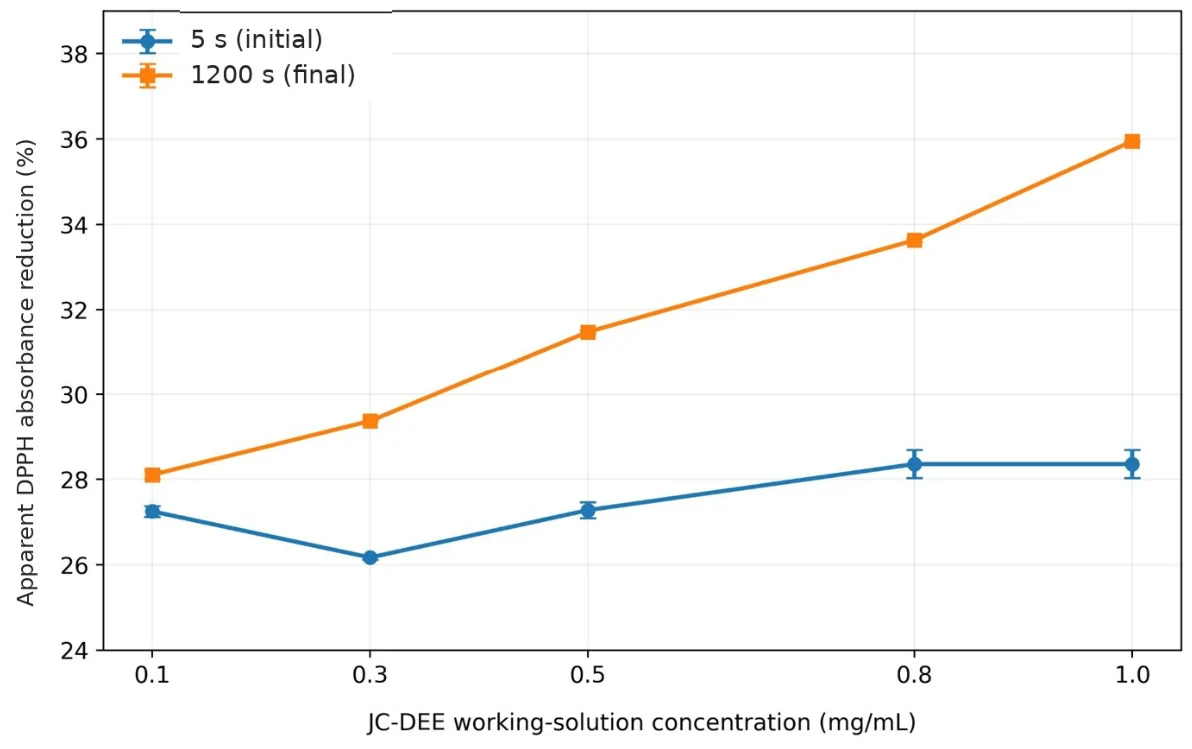
Exploratory, unvalidated DPPH spectrophotometric responses of JC-DEE at 5 s and 1200 s, expressed as apparent absorbance reduction relative to the unmatched DPPH reference control. Data are presented as mean ± SD (*n* = 3 within-day determinations). Concentrations correspond to the prepared JC-DEE solutions before addition to the DPPH reagent. The observed variations reflect uncorrected spectrophotometric measurements and cannot be attributed exclusively to DPPH radical scavenging because of the unmatched reference control and the absence of sample-specific blanks.

At the initial measurement (5 s), the apparent absorbance reduction values ranged from 26.17% to 28.36% across the five tested JC-DEE concentrations, with no consistent concentration-associated pattern. At 1200 s, the calculated indices ranged from 28.11% to 35.95%, with progressively higher values observed at increasing extract concentrations (Table 5, Figure 2). The ascorbic acid reference exhibited apparent absorbance reduction values of 84.98% and 86.74% at 5 s and 1200 s, respectively. These values are reported descriptively and are not intended for quantitative comparison of antioxidant potency.

The apparent reductions were calculated using an undiluted DPPH reference control, whereas the sample-containing mixtures had a lower final DPPH concentration as a consequence of the experimental mixing procedure. In addition, the intrinsic absorbance of JC-DEE at 517 nm could not be independently corrected. Therefore, the recorded differences may reflect a combination of DPPH dilution, sample-dependent optical contributions, and possible chemical interactions within the reaction mixtures. The relative contributions of these factors cannot be determined from the available measurements.

Accordingly, the concentration- and time-associated variations illustrated in Table 5 and Figure 2 are presented solely as descriptive, unvalidated experimental observations. They do not establish quantitative antioxidant activity, radical-scavenging potency, or a validated concentration–response relationship. Additional measurements using volume- and solvent-matched controls and extract-specific blanks would be necessary to determine the actual radical-scavenging capacity of JC-DEE.

### 3.5. Antimicrobial Activity

JC-DEE showed measurable antimicrobial activity against the tested reference microorganisms under the applied disk-diffusion conditions (Table 6). The largest inhibition zones were recorded for *S. pyogenes* and *S. aureus* (10 mm), followed by *E. coli* and *P. aeruginosa* (8 mm). The lowest response was observed for *C. parapsilosis*, with an inhibition zone of 7 mm. Since none of the tested microorganisms produced an inhibition zone exceeding the predefined screening threshold of 15 mm, MIC determination was not performed.

### 3.6. Effect on Cell Viability

The effect of JC-DEE on cell viability was evaluated in non-malignant HaCaT keratinocytes and A375 melanoma cells after 24 h of exposure to concentrations ranging from 25 to 300 μg/mL. JC-DEE produced a concentration-dependent reduction in cell viability in both cell lines, although the magnitude of the response differed across the tested concentration range.

**Figure 3:**
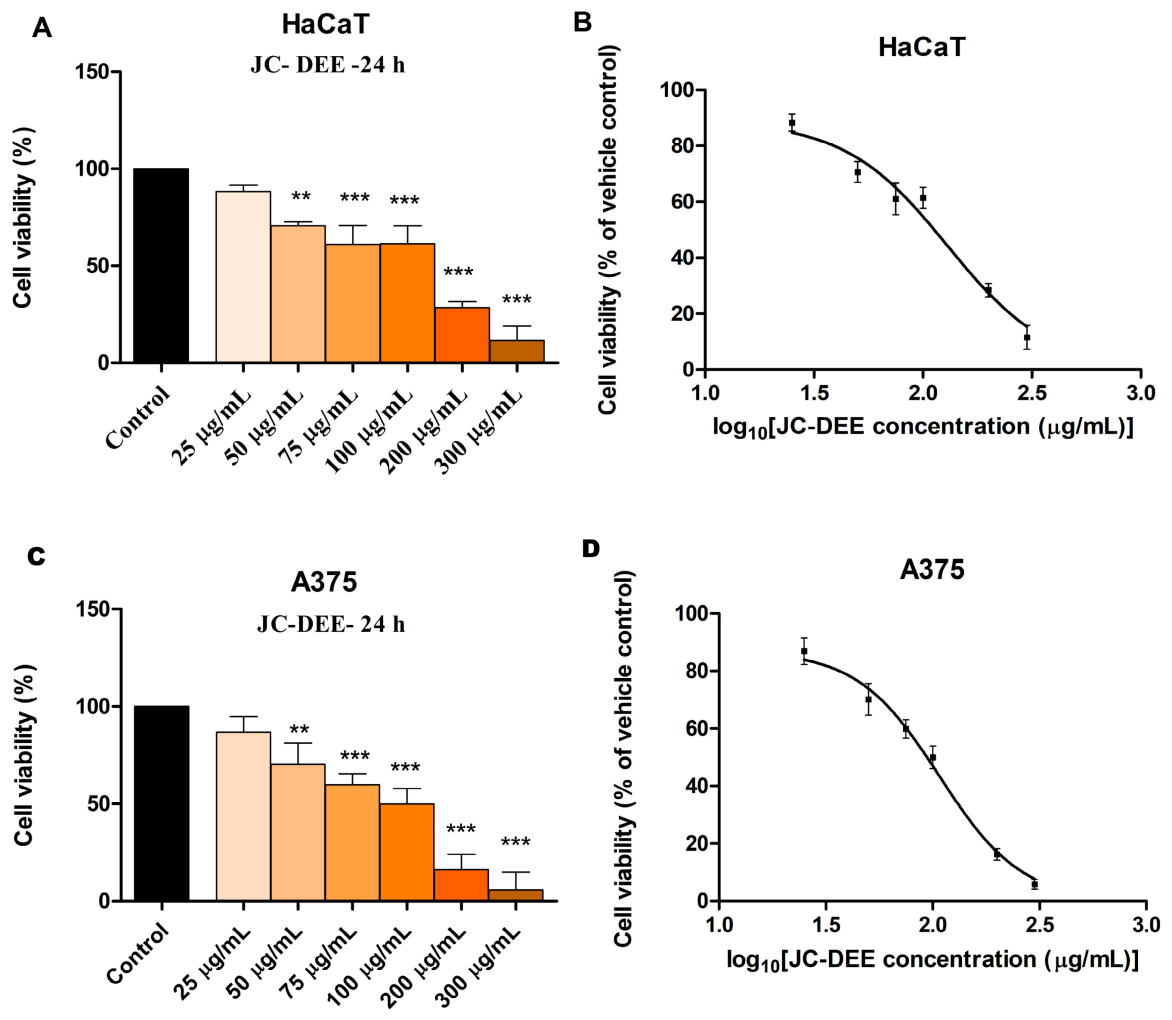
Effect of JC-DEE on the viability of HaCaT and A375 cells after 24 h of treatment. Panels (**A**,**C**) show the viability results for HaCaT and A375 cells; panels (**B**,**D**) show the corresponding concentration–response data and nonlinear regression fits. Cells were exposed to JC-DEE concentrations ranging from 25 to 300 μg/mL. Cell viability was determined using the Alamar Blue assay and expressed as a percentage relative to the corresponding vehicle control containing 0.5% DMSO (100%). Data are presented as mean ± SD of three independent experiments performed in triplicate (*n* = 3 biological replicates, each with *n* = 3 technical replicates) ** *p* < 0.01, and *** *p* < 0.001 versus control.

In HaCaT cells, a significant reduction in viability was observed from 50 μg/mL onward. Cell viability decreased to 70.63 ± 2.0% at 50 μg/mL, 61.03 ± 9.7% at 75 μg/mL, and 61.39 ± 9.3% at 100 μg/mL. A more pronounced decrease occurred at the two highest concentrations, with viability reaching 28.44 ± 3.1% at 200 μg/mL and 11.56 ± 7.3% at 300 μg/mL (Figure 3).

A375 melanoma cells showed a similar concentration-dependent response to JC-DEE. The reduction in viability became statistically significant at 50 μg/mL and progressively increased with concentration. At 200 and 300 μg/mL, cell viability decreased to 16.20 ± 7.8% and 5.79 ± 3.1%, respectively (Figure 3).

Nonlinear concentration–response analysis yielded an estimated IC_50_ value of 102.7 μg/mL (95% CI: 67.9–155.1 µg/mL) for A375 cells and 129.3 μg/mL (95% CI: 68–247 µg/mL) for HaCaT cells. Although the calculated IC_50_ value was numerically lower for A375 cells, the findings only show a limited difference in the cytotoxic response and do not establish a selective activity against melanoma cells. Moreover, as Alamar Blue assay assessed cell viability, the observed reductions primarily reflect decreased cellular metabolic activity and do not establish cell death.

### 3.7. Cell Morphology

**Figure 4:**
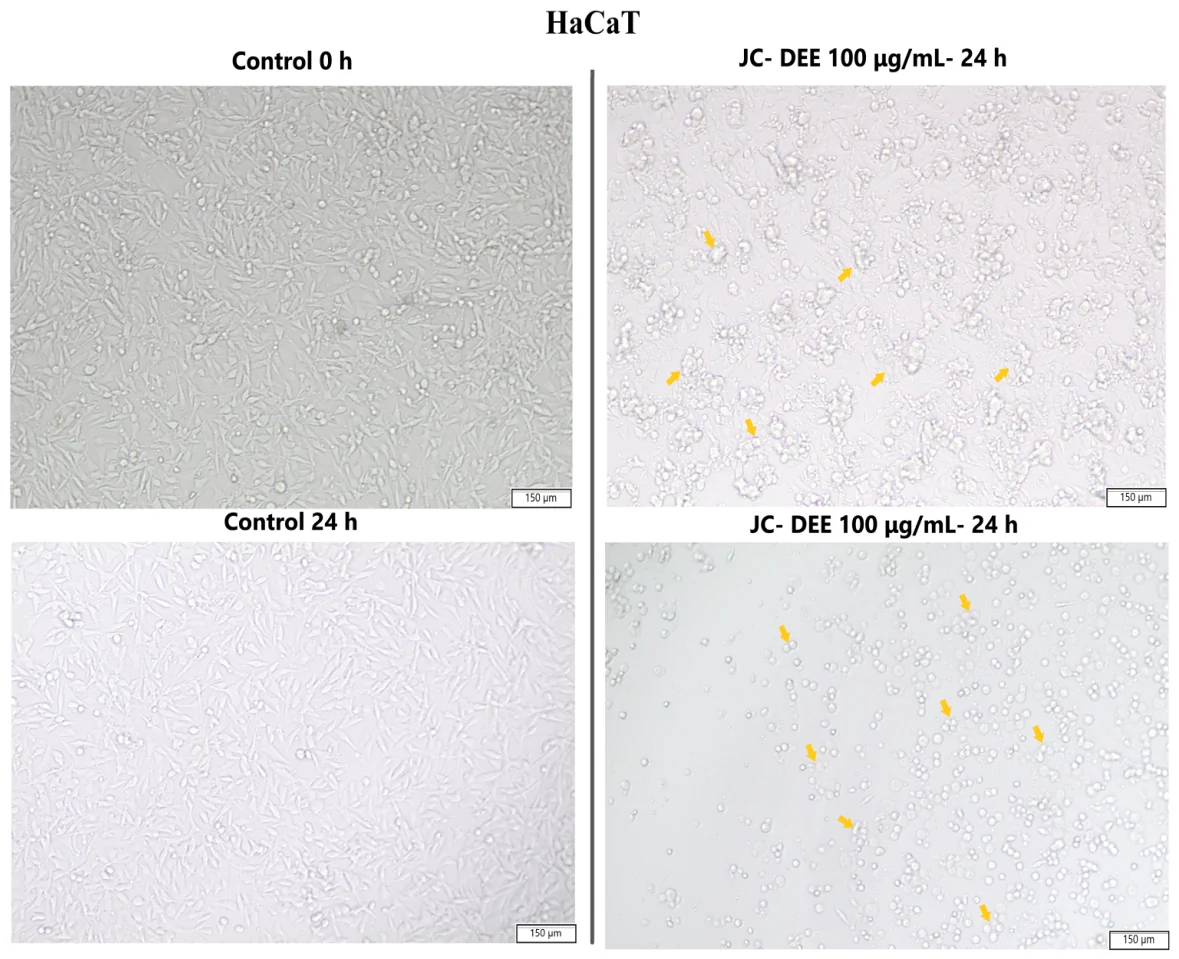
Representative phase-contrast microscopy images of HaCaT cells. Untreated control cells are shown at 0 and 24 h. JC-DEE-treated cells are shown after 24 h of exposure to concentrations of 100 and 300 µg/mL, as indicated in the panel labels. Yellow arrows highlight representative morphological alterations, including cell rounding, decreased confluence, cellular shrinkage, and partial cell detachment. Images were obtained using an inverted microscope. Scale bar = 150 µm.

Morphological examination by phase-contrast microscopy supported the concentration-dependent effects observed in the cell viability assay. In HaCaT cells, exposure to JC-DEE for 24 h induced alterations in cell morphology at both 100 and 300 μg/mL, characterized by cell rounding, loss of adherence, detachment, and reduced cell density. These alterations were more pronounced at the higher concentration (Figure 4). The experimental material reports these features for the diethyl ether fraction together with the other more cytotoxic fractions.

**Figure 5:**
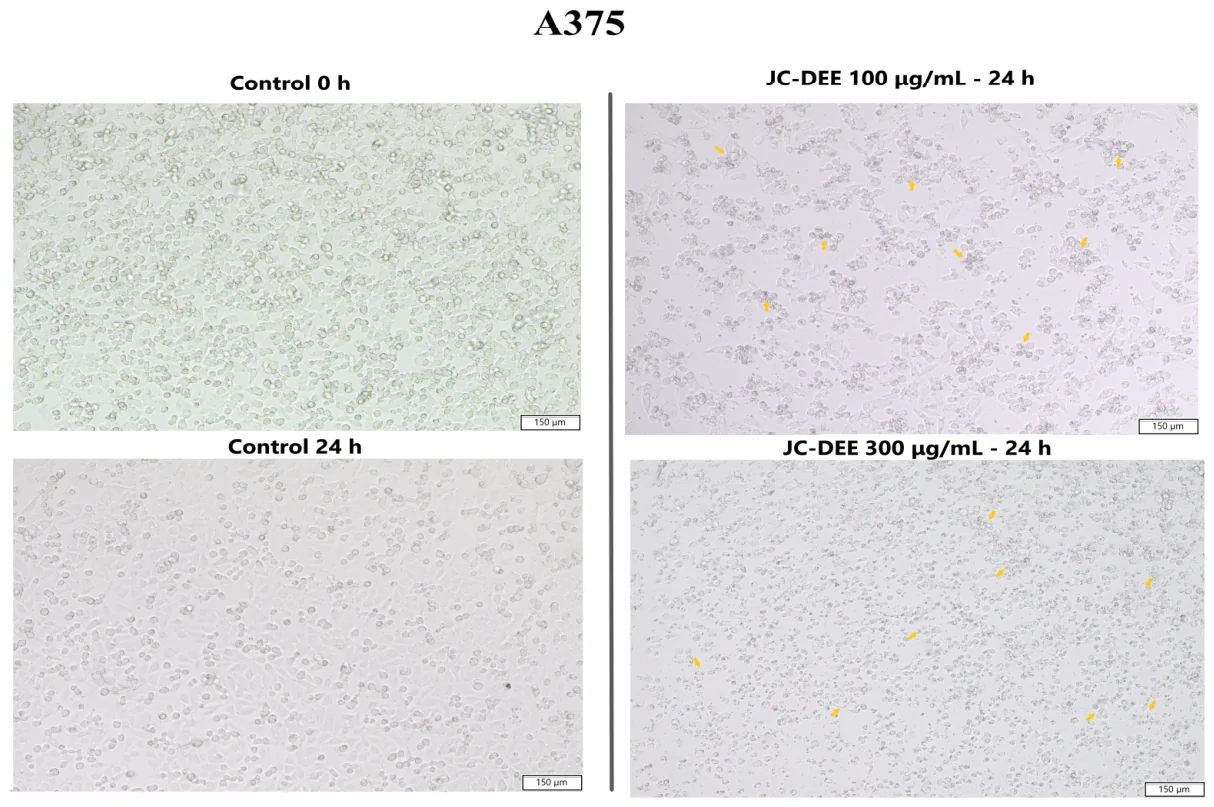
Representative phase-contrast microscopy images of A375 cells. Untreated control cells are shown at 0 and 24 h. JC-DEE-treated cells are shown after 24 h of exposure to concentrations of 100 and 300 µg/mL, as indicated in the panel labels. Yellow arrows highlight representative morphological alterations, including cell rounding, decreased confluence, cellular shrinkage, and partial cell detachment. Images were obtained using an inverted microscope. Scale bar = 150 µm.

A375 cells exhibited more obvious morphological alterations following JC-DEE treatment, compared to HaCaT cells. At 100 μg/mL, changes in cell shape, rounding, shrinkage, loss of adhesion, and reduced cell density were apparent, whereas exposure to 300 μg/mL resulted in more extensive cellular damage, including pronounced detachment and fragmentation. The progressive morphological alterations were consistent with the reduction in cell viability observed in the Alamar Blue assay (Figure 5).

**Figure 6:**
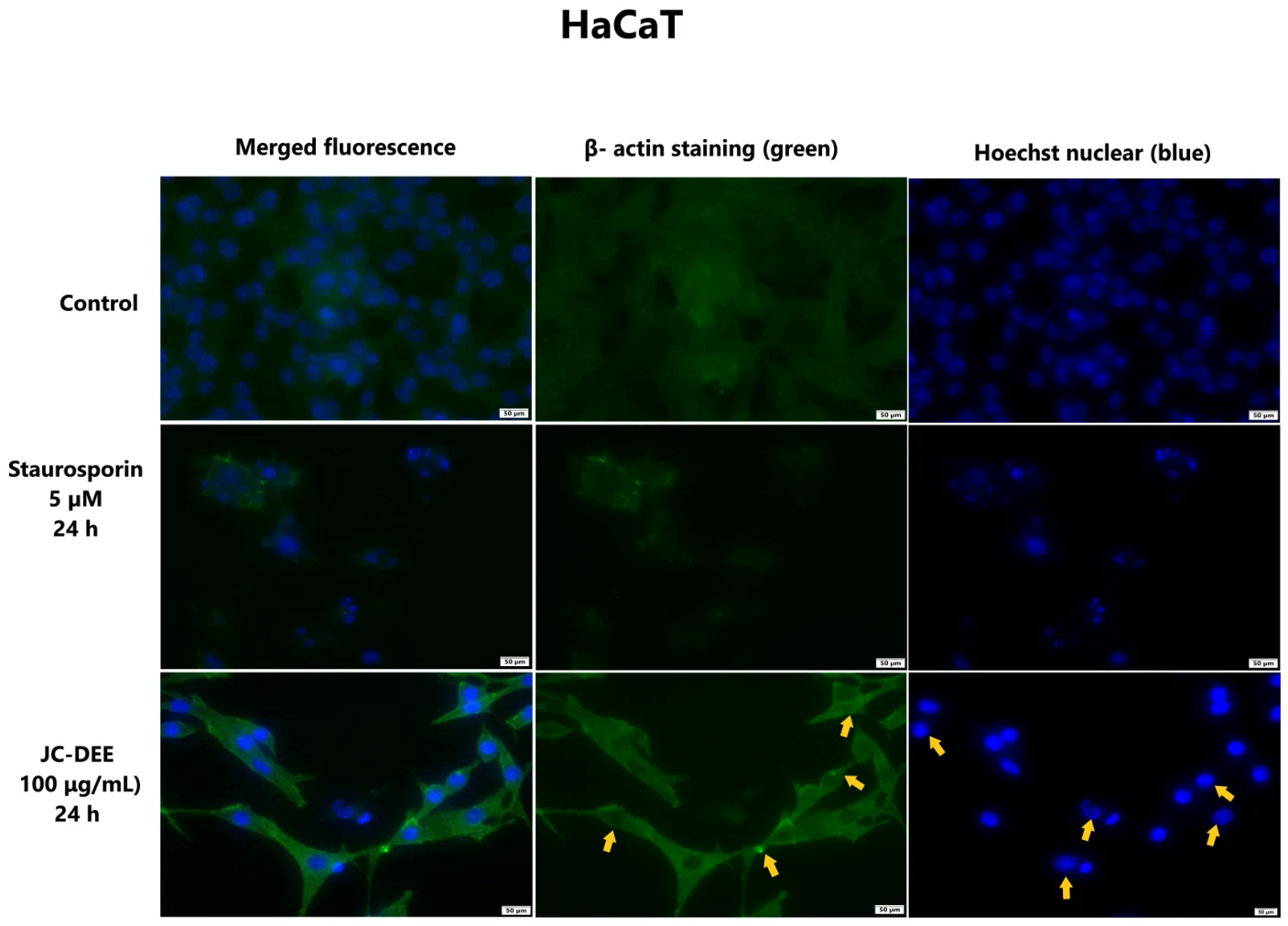
Effects of 24 h treatment with JC-DEE (100 µg/mL) on HaCaT cell nuclear morphology (Hoechst 33342, blue) and β-actin staining patterns (green). Staurosporine (2.5 µM, 24 h) was used as a positive control for apoptosis induction. The columns show merged images, β-actin staining, and Hoechst staining, respectively. Yellow arrows indicate alterations in β-actin staining patterns and nuclear morphological changes suggestive of apoptosis. Scale bar = 50 µm.

Immunofluorescence analysis was performed after 24 h of exposure to 100 μg/mL JC-DEE to further examine the cytoskeletal organization and nuclear morphology. HaCaT cells exposed to JC-DEE treatment showed lower cell density without severe alterations in β-actin organization. Even though most nuclei remained intact, occasionally intensely stained nuclei reflecting chromatin condensation, nuclear shrinkage, and in some cases, fragmentation were noted, compared with untreated cells (Figure 6).

**Figure 7:**
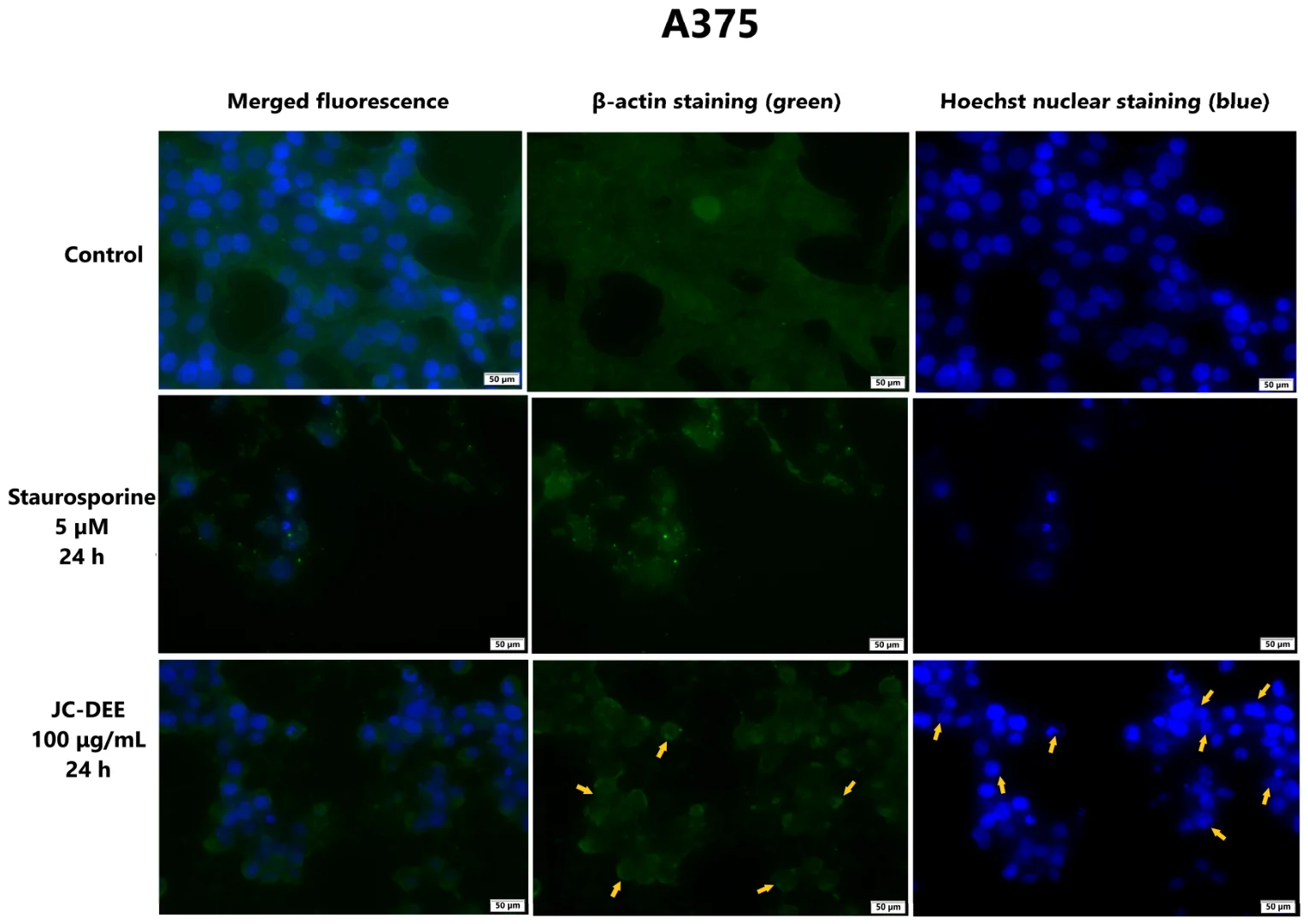
Effects of 24 h treatment with JC-DEE (100 µg/mL) on A375 cell nuclear morphology (Hoechst 33342, blue) and β-actin staining patterns (green). Staurosporine (2.5 µM, 24 h) was used as a positive control for apoptosis induction. The columns show merged images, β-actin staining, and Hoechst staining, respectively. Yellow arrows indicate alterations in β-actin staining patterns and nuclear morphological changes suggestive of apoptosis. Scale bar = 50 µm.

**Figure 8:**
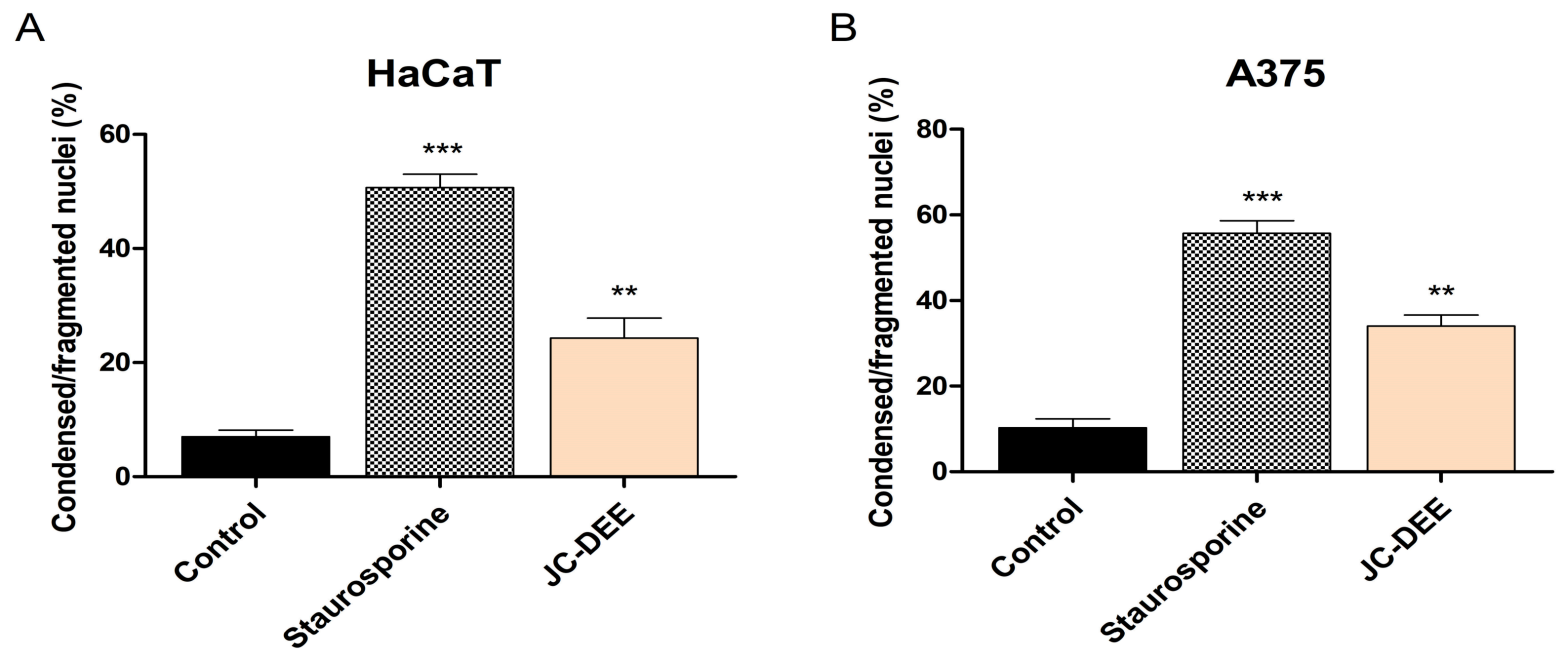
The quantification of the nuclear morphological changes in (**A**) HaCaT and (**B**) A375 cell lines following the 24 h treatment with 100 μg/mL JC-DEE. The cells displaying condensed and/or fragmented nuclei were manually counted in five randomly selected fields per condition (subsamples). Data are expressed as mean ± SD of three independent experiments (** *p* < 0.01, and *** *p* < 0.001).

In comparison to the HaCaT cell line, exposure of A375 melanoma cells to JC-DEE was associated with pronounced cytoskeletal reorganization and loss of normal cellular architecture. The observed nuclear alterations included chromatin condensation, nuclear shrinkage, and fragmentation, together with small chromatin fragments morphologically resembling apoptotic bodies (Figure 7 and Figure 8). These findings demonstrate marked JC-DEE-induced alterations in both cytoskeletal organization and nuclear morphology in A375 cells, and more severe than those observed in HaCaT cells.

## 4. Discussion

The present study provides an integrated phytochemical and biological characterization of the diethyl ether fraction obtained from *Juniperus communis* L. pseudo-fruits. Targeted HPLC-MS analysis identified selected phenolic constituents, while FTIR spectroscopy provided complementary information on the functional groups present in the fraction. Biological evaluation revealed measurable antimicrobial effects and a concentration-dependent reduction in the viability of both A375 melanoma cells and HaCaT keratinocytes, while the exploratory DPPH assessment provided preliminary, unvalidated spectrophotometric observations. Morphological and immunofluorescence analyses further demonstrated cellular, cytoskeletal, and nuclear alterations following JC-DEE exposure. Collectively, these findings characterize JC-DEE as a chemically and biologically active fraction, while also indicating that its cytotoxicity is not restricted to melanoma cells.

### 4.1. Phytochemical Profile and Total Phenolic Content

The JC-DEE spectrum is consistent with a mixture containing aliphatic groups, oxygenated functions, and unsaturated constituents; the 1508.33 cm^−1^ band also supports a possible aromatic contribution. The intense 1697.36 cm^−1^ absorption supports carbonyl functionality, but assigning a specific ketone, aldehyde, ester, or acid solely from this mixed spectrum would be unwarranted. Likewise, the C–O and low-wavenumber bands cannot establish the identities or substitution patterns of phenolic compounds without complementary data. Moisture from KBr and atmospheric CO_2_ may affect parts of the spectrum.

A published study of an aqueous *J. communis* leaf extract reported O–H, aliphatic C–H, and C–O features [36]. This broad similarity concerns functional groups only because the extraction procedure and measurement mode differed. Recent LC-MS analysis of hydroalcoholic *J. communis* extracts has also confirmed the presence of a chemically diverse secondary-metabolite profile, although direct quantitative comparison with JC-DEE is limited by differences in extraction polarity and sample preparation [37].

Targeted HPLC–MS analysis of JC-DEE showed that apigenin (1.971 ± 0.150 µg/mL), protocatechuic acid (1.070 ± 0.049 µg/mL), and luteolin (0.560 ± 0.046 µg/mL) were the most abundant compounds among the quantified analytes, whereas the other quantified constituents were present at lower concentrations. These findings indicate the presence of selected flavonoids and phenolic acids in the diethyl ether fraction without establishing their relative contribution to its overall chemical composition. The total phenolic content (TPC) determined using the Folin–Ciocalteu assay and the concentrations obtained by targeted HPLC–MS represent distinct analytical measurements and should not be directly equated. The Folin–Ciocalteu assay measures the reducing capacity of the sample under the experimental conditions, expressed as chlorogenic acid equivalents (CAE), and may also respond to non-phenolic reducing substances. In contrast, targeted HPLC–MS provides compound-specific quantitative information for a defined set of analytes. Consequently, the TPC value should not be interpreted as the actual mass of phenolic compounds present in JC-DEE, nor should it be expected to correspond directly to the sum of the individually quantified HPLC–MS constituents.

These findings are consistent with previous phytochemical investigations of *J. communis*. Tang et al. [38] demonstrated a complex polyphenolic composition of juniper berries, comprising several classes of phenolic acids and flavonoids, and identified protocatechuic acid as the predominant phenolic acid in the analyzed berries. Mërtiri et al. [39] further confirmed the occurrence of protocatechuic acid in *J. communis* berry extracts and luteolin in needle-leaf extracts. More recently, Büker et al. [37] identified protocatechuic acid, apigenin, and luteolin among the major phenolic constituents of *J. communis* extracts, providing additional support for the occurrence of the three predominant compounds detected in JC-DEE. Differences in their relative abundance among studies may reflect variations in plant material, geographical origin, extraction procedures, and analytical conditions. Beyond the predominant constituents, JC-DEE contained several additional phenolic acids and flavonoid derivatives that further illustrate the phytochemical diversity of the fraction. Notably, apigenin-7-O-glucoside and kaempferol-3-O-glucoside have previously been identified in *J. communis* berry extracts, supporting their occurrence in the same plant material investigated here [40]. Several of the minor phenolic acids detected in JC-DEE, including gallic, vanillic, *p*-coumaric, and ferulic acids, have also been reported in *J. communis* or other *Juniperus* matrices [38,39]. Of particular interest, umbelliferone has previously been reported in *J. communis* [7,41] and has also been identified in other members of the genus *Juniperus*. Orhan et al. identified and quantified this coumarin in leaf extracts of *J. foetidissima* and *J. sabina*, further supporting its occurrence within the genus [42]. Acacetin was also detected as a minor constituent of JC-DEE; however, its occurrence appears to be less extensively documented in *Juniperus*. The occurrence of these minor constituents alongside apigenin, protocatechuic acid, and luteolin therefore broadens the phytochemical characterization of JC-DEE and highlights the chemical diversity retained in this low-polarity fraction.

The total phenolic content of JC-DEE was 114.22 ± 5.35 mg chlorogenic acid equivalents/g fraction, demonstrating a substantial content of Folin-Ciocâlteu-reactive constituents. Previous investigations have demonstrated substantial variability in the phenolic composition of *J. communis* pseudo-fruits. Popescu et al. [43] examined hydroalcoholic extracts prepared from pseudo-fruits collected from different Romanian geographical areas and at different harvest times and identified 15 phenolic compounds belonging to phenolic acid and flavonoid classes. The authors also demonstrated significant variation in total polyphenol content according to geographical origin and harvesting period [43]. Similarly, Miceli et al. [44] reported differences in flavonoid composition and biological properties between berry extracts of *J. communis* var. *communis* and *J. communis* var. *saxatilis*. These studies demonstrate that the phytochemical profile of *J. communis* berries varies with botanical material, geographical origin, harvesting conditions, and extraction procedure, factors that should be considered when comparing different preparations [44].

### 4.2. Exploratory DPPH Findings and Antioxidant Potential

The preliminary DPPH investigation of JC-DEE yielded spectrophotometric responses that varied according to sample concentration and measurement time. The apparent absorbance reduction indices exhibited a relatively narrow distribution at the initial measurement, whereas greater differentiation between the tested concentrations was observed after 1200 s. Although these observations describe changes in the measured reaction mixtures, their interpretation is constrained by methodological factors that prevent reliable attribution of the recorded responses to radical-scavenging activity. Specifically, the experimental design employed a DPPH reference control that was not matched to the sample-containing mixtures in terms of final volume and solvent composition. The addition of 0.3 mL of sample solution to 2.7 mL of DPPH reagent reduced the nominal DPPH concentration from 0.10 mM to 0.09 mM, potentially introducing an apparent decrease in absorbance independently of radical scavenging. Moreover, the absence of extract-specific blanks prevented correction for the intrinsic absorbance of JC-DEE at the analytical wavelength of 517 nm. These sources of interference cannot be adequately separated or quantified retrospectively using the available experimental data. Consequently, the numerical values reported should be regarded exclusively as unvalidated, descriptive spectrophotometric indices. Neither their absolute magnitude nor their apparent concentration- and time-associated variation provides sufficient evidence for establishing the antioxidant potency of JC-DEE. In particular, the observed differences cannot be interpreted as demonstrating reaction kinetics attributable specifically to DPPH radical scavenging.

Nevertheless, the investigation of the antioxidant potential of JC-DEE remains relevant in view of its phytochemical composition. Targeted HPLC–MS analysis confirmed the presence of several phenolic constituents, including apigenin, protocatechuic acid, and luteolin, while the Folin–Ciocâlteu assay indicated a total phenolic content of 114.22 ± 5.35 mg chlorogenic acid equivalents/g fraction. These independently obtained analytical findings provide a compositional basis for further investigation of the redox-related properties of JC-DEE, without constituting direct evidence of its antioxidant activity.

Previous studies have documented antioxidant properties in preparations obtained from *Juniperus communis* cone berries, using different extraction procedures and analytical approaches [38,44]. However, differences in phytochemical composition, extraction conditions, and experimental methodologies preclude direct extrapolation of these findings to the present diethyl ether fraction. Likewise, the antioxidant properties reported for individual phenolic constituents cannot be assumed to represent the activity of the complete JC-DEE mixture. Therefore, the present DPPH observations are retained as preliminary experimental findings for methodological transparency and to document the spectrophotometric behavior recorded under the original assay conditions. Confirmation of the antioxidant capacity of JC-DEE will require further investigation using appropriately matched controls, sample-specific blanks, and complementary antioxidant assays. Until such validation is available, no quantitative conclusions regarding the radical-scavenging activity or antioxidant efficacy of JC-DEE can be established from the present DPPH measurements.

### 4.3. Antimicrobial Effects

The antimicrobial evaluation of JC-DEE revealed limited growth inhibition under the disk-diffusion conditions employed. The largest inhibition zones were recorded for *S. pyogenes* and *S. aureus* (10 mm), followed by *E. coli* and *P. aeruginosa* (8 mm), whereas *C. parapsilosis* exhibited the smallest inhibition zone (7 mm). None of the tested microorganisms produced an inhibition zone exceeding the predefined screening threshold of 15 mm; consequently, MIC determination was not performed, representing a screening limitation of the present study. However, this threshold represents an operational criterion adopted for preliminary antimicrobial screening rather than an established susceptibility breakpoint. Importantly, inhibition zones below this threshold do not necessarily indicate the absence of antimicrobial activity, as disk-diffusion results may be influenced by the solubility and diffusion characteristics of the tested constituents. Therefore, the present findings should be interpreted as evidence of limited inhibitory effects under the specific experimental conditions, while additional broth-dilution assays would be required to characterize the antimicrobial potency of JC-DEE more comprehensively.

The microorganism-dependent response observed here is consistent with the variability reported for other *J. communis* pseudo-fruit preparations. Popescu et al. evaluated hydroalcoholic pseudo-fruit extracts against Gram-positive and Gram-negative bacterial strains and demonstrated that antibacterial activity varied according to bacterial species, geographical origin of the plant material, and harvest time. Their study reported activity against all tested Gram-positive strains, whereas susceptibility among the investigated Gram-negative bacteria was less consistent. However, the microbial panel used by Popescu et al. [43] differed from that of the present study, and *S. pyogenes* was not included. Therefore, their results support variability in bacterial susceptibility to *J. communis* extracts but should not be interpreted as a direct comparison with the response of *S. pyogenes* observed for JC-DEE.

Miceli et al. also demonstrated antimicrobial activity in methanolic berry extracts of *J. communis* and reported differences between botanical varieties [44]. Together with the present findings, these data indicate that antimicrobial activity cannot be generalized across *J. communis* preparations because the chemical composition of crude extracts and solvent-derived fractions differs considerably. The small inhibition zones observed for JC-DEE thus provide preliminary evidence of its antimicrobial behavior under the specific disk-diffusion conditions employed.

### 4.4. Effects on Cell Viability

JC-DEE produced a clear concentration-dependent reduction in cell viability after 24 h of exposure in both A375 melanoma cells and HaCaT keratinocytes. In A375 cells, the effect became particularly pronounced at the highest concentrations, with viability decreasing to 16.20 ± 7.8% at 200 μg/mL and 5.79 ± 3.1% at 300 μg/mL. The cytotoxic response was somewhat more pronounced in A375 cells than in HaCaT keratinocytes.

The estimated IC_50_ values provide a quantitative indication of the response of the two cellular models to JC-DEE. Nonlinear regression analysis using a variable-slope logistic model yielded an estimated IC_50_ value of 102.7 μg/mL (95% CI: 67.9–155.1 µg/mL) for A375 cells and 129.3 μg/mL (95% CI: 68–247 µg/mL) for HaCaT cells, corresponding to an approximately 1.26-fold difference. An important limitation of this study, that must be considered when interpreting the results, is the use of HaCaT cells as a non-malignant comparator. Specifically, although its widespread used as an experimental model, the immortalized HaCaT keratinocyte cell line differs from melanocytes in their biological properties and origin and does not represent the normal counterpart of malignant melanoma A375 cell line. Consequently, the comparison between the two lines cannot establish melanoma-specific cytotoxicity and the relatively close IC_50_ values indicate a limited differential cytotoxicity under our experimental conditions.

With regard to the possible relationship between the cytotoxic effects of JC-DEE and its chemical composition, targeted HPLC-MS analysis revealed that apigenin (1.971 ± 0.150 µg/mL), protocatechuic acid (1.070 ± 0.049 µg/mL), and luteolin (0.560 ± 0.046 µg/mL) were the most abundant among the quantified phenolic constituents.

Protocatechuic acid (3,4-dihydroxybenzoic acid) is a naturally occurring hydroxybenzoic acid widely distributed in plants and has been investigated for antioxidant, anti-inflammatory, and antiproliferative properties [45]. Its relevance to melanoma has previously been investigated in the context of melanogenesis. Truong et al. [46] demonstrated that protocatechuic acid isolated from pear inhibited melanin production and cellular tyrosinase activity in α-MSH-stimulated melanoma cells. The observed effects were associated with decreased expression of melanogenic enzymes and microphthalmia-associated transcription factor (MITF), together with modulation of the cAMP/CREB pathway [46]. Protocatechuic acid and some of its derivatives have also been investigated for antioxidant and melanogenesis-modulating effects in melanoma-derived cell models; however, these findings do not directly establish cytotoxic or anti-melanoma activity [47]. Lin et al. demonstrated in AGS gastric carcinoma cells that protocatechuic acid inhibited migration and invasion at non-cytotoxic concentrations by reducing MMP-2 expression and modulating RhoB/PKCε and Ras/Akt/NF-κB signaling. Importantly, its anti-metastatic activity was also evaluated in vivo using B16/F10 melanoma cells, where oral administration of protocatechuic acid reduced hepatic metastatic colonization in mice [48]. Flavonoids have also been extensively investigated for their ability to affect cell proliferation, apoptosis, oxidative stress, migration, invasion, and intracellular signaling. Luteolin, a naturally occurring flavone found in numerous medicinal and edible plants, has emerged as a promising bioactive compound in this context. Its anticancer effects have been associated with the modulation of multiple signaling pathways, suggesting that its biological activity may extend beyond a single molecular target [49]. More recently, Hussein et al. [50] investigated luteolin in the human A375 cutaneous melanoma cell line and reported inhibition of proliferation and migration, induction of apoptosis, and cell-cycle arrest. Their findings further implicated G-protein-coupled estrogen receptor (GPER)-associated signaling and changes in P-ERK and c-Myc expression in the response of A375 cells to luteolin [50]. These observations are particularly relevant when considering plant extracts in which luteolin is present as one of several potentially bioactive constituents. Yao et al. demonstrated that purified luteolin inhibited A375 proliferation and induced apoptosis in a concentration-dependent manner, while also suppressing migration and invasion. These effects were associated with inhibition of PI3K/AKT signaling, decreased MMP-2 and MMP-9 expression, and increased TIMP-1 and TIMP-2; inhibition of A375-derived tumor growth was additionally demonstrated in a xenograft model [51]. More recent evidence further supports the melanoma-related activity of luteolin: Li et al. [52] showed that luteolin induced apoptosis and suppressed migration and invasion in A375 and B16F10 cells through inhibition of Src/STAT3 signaling and promotion of STAT3 degradation, with corresponding antitumor effects in melanoma-bearing mice. The targeted HPLC–MS analysis identified apigenin, protocatechuic acid, and luteolin as the most abundant compounds among the quantified analytes in JC-DEE, with apigenin exhibiting the highest measured concentration. Previous investigations have demonstrated that luteolin exerts anti-melanoma effects in experimental models. In particular, Li et al. [52] reported that luteolin reduced melanoma cell viability and modulated Src/STAT3 signaling in both cellular and animal models. However, the contribution of luteolin or other identified constituents to the effects observed for JC-DEE cannot be established from the present experimental data. Moreover, the targeted analytical approach does not provide a comprehensive characterization of the fraction’s chemical composition.

Hasnat et al. demonstrated in A375 and A2058 human melanoma cells that apigenin reduced cell proliferation and viability in a time- and concentration-dependent manner. Apigenin also induced morphological alterations, reduced integrin protein levels, inhibited FAK and ERK1/2 phosphorylation, and increased caspase-3 and cleaved PARP levels, supporting the induction of anoikis-associated apoptotic signaling [53]. Woo et al. subsequently demonstrated in A375P and A375SM melanoma cells that apigenin caused a concentration-dependent reduction in viability and increased chromatin condensation; importantly, apoptosis was confirmed by Annexin V/propidium iodide staining and changes in apoptosis-associated proteins, with modulation of Akt and MAPK pathways [54]. These observations are relevant to the phenotype observed with JC-DEE, particularly the loss of adhesion and nuclear alterations, but they cannot be extrapolated mechanistically to the fraction.

The relatively low concentrations of the individually quantified phenolics suggest that the cytotoxicity of JC-DEE cannot be attributed solely to protocatechuic acid, luteolin, or apigenin. The observed activity may reflect the combined contribution of multiple constituents, including compounds not captured by the targeted HPLC-MS analysis. Potential additive, synergistic, or antagonistic interactions within the fraction remain hypothetical and require direct experimental investigation. Accordingly, JC-DEE should be considered a complex bioactive fraction, and its cellular effects should not be attributed to any single identified phenolic compound.

### 4.5. Cellular Morphology and Immunofluorescence Analysis

The viability data were supported by marked morphological alterations following JC-DEE exposure. Phase-contrast microscopy showed rounding, loss of adherence, detachment, and reduced cell density in HaCaT cells treated with JC-DEE, with these alterations becoming more pronounced at the higher concentration. A375 cells exhibited similar but extensive structural changes, including rounding, shrinkage, fragmentation, loss of adhesion, detachment, and a marked reduction in cell density. The agreement between the Alamar Blue results and the microscopic observations strengthens the evidence that JC-DEE compromises cellular viability and structural integrity rather than producing an isolated change in metabolic readout.

Loss of adhesion is particularly relevant in melanoma biology because survival of detached malignant cells requires resistance to anoikis, a form of programmed cell death triggered by loss of appropriate cell–extracellular matrix attachment. In A375 and A2058 human melanoma cells, Hasnat et al. demonstrated that apigenin reduced integrin protein levels and inhibited FAK and ERK1/2 phosphorylation. These effects were accompanied by morphological alterations and reduced cell migration, while increased caspase-3 and cleaved PARP levels in detached cells supported anoikis-associated apoptotic signaling [53]. The loss of adherence and detachment observed after JC-DEE treatment are therefore phenotypically compatible with altered adhesion-dependent survival. However, because integrins, FAK/ERK signaling, caspases, and PARP were not evaluated in the present study, neither anoikis nor the corresponding signaling pathway can be assigned as a mechanism of JC-DEE activity. Immunofluorescence analysis further demonstrated that the effect of JC-DEE extended to both cytoskeletal organization and nuclear architecture. At 100 μg/mL, HaCaT cells showed reduced cell density with comparatively preserved β-actin organization, while occasional nuclei displayed chromatin condensation, shrinkage, or fragmentation. In A375 cells, JC-DEE produced pronounced disruption of cytoskeletal organization and loss of structural integrity, while Hoechst staining revealed condensed chromatin, nuclear shrinkage and fragmentation, together with small condensed chromatin structures resembling apoptotic bodies. These findings provide morphological evidence that the reduction in metabolic viability is accompanied by substantial structural injury at both cytoplasmic and nuclear levels.

The nuclear phenotype observed in JC-DEE-treated A375 cells is consistent with morphological features associated with apoptosis. However, these results do not independently confirm apoptosis and further studies using specific apoptosis assays are required to confirm apoptosis induction and to elucidate the underlying molecular mechanism. Woo et al. demonstrated in A375P and A375SM melanoma cells that apigenin induced concentration-dependent chromatin condensation, while apoptosis was independently confirmed by Annexin V/propidium iodide staining and changes in apoptosis-associated proteins. The authors also demonstrated modulation of Akt and MAPK signaling [54]. Similarly, Yao et al. demonstrated that luteolin inhibited proliferation and induced apoptosis in A375 cells, together with modulation of MMP-2/MMP-9 and PI3K/AKT signaling [51]. In contrast, the present study assessed nuclear morphology by Hoechst staining and cytoskeletal organization by β-actin immunofluorescence but did not evaluate phosphatidylserine externalization, caspase activation, PARP cleavage, or other apoptosis-specific biochemical endpoints. Therefore, the observed chromatin condensation, nuclear fragmentation, and apoptotic body-like structures should be interpreted as an apoptosis-compatible morphological phenotype rather than definitive evidence that apoptosis represents the mechanisms underlying the observed cellular alterations.

## 5. Conclusions

This study provides an integrated phytochemical and biological characterization of a diethyl ether fraction obtained from *Juniperus communis* L. cone berries. Targeted HPLC–MS analysis identified apigenin, protocatechuic acid, and luteolin as the most abundant compounds among the quantified analytes, together with several other phenolic acids and flavonoid derivatives. JC-DEE produced limited growth inhibition against the tested microorganisms under the disk-diffusion conditions employed. Although exploratory DPPH measurements revealed apparent spectrophotometric responses, their methodological limitations precluded validation of the fraction’s radical-scavenging activity. These findings contribute to the characterization of the phytochemical composition and biological properties of this fraction.

The cellular assays revealed that JC-DEE reduces, in a concentration-dependent manner, the metabolic activity of A375 cells, accompanied by pronounced morphological, cytoskeletal, and nuclear alterations. Comparable effects were also observed in HaCaT keratinocytes. Although several of these changes suggest that JC-DEE induced apoptosis-like morphological changes, further studies using biochemical and molecular assays are warranted to confirm the underlying mechanisms of cell death.

Overall, these findings provide preliminary evidence of the in vitro cytotoxic effect of JC-DEE in established cellular models. Future studies should clarify the contribution of its major phytochemical constituents, establish the mechanisms underlying its cellular effects, and evaluate its cytotoxic selectivity in additional counterpart non-malignant cells. Further investigations employing appropriately controlled and validated antioxidant assays are also warranted to establish whether the phytochemical composition of JC-DEE translates into measurable radical-scavenging activity.

## Figures and Tables

**Table 1:** Targeted LC-HRMS(/MS) quantification of phenolic compounds confirmed with authentic reference standards—chromatographic and detection details.

No.	Compound Name	Molecular Formula	Retention Time (min)	Quantitation Mode	Precursor Ion *m*/*z* *
1	Gallic acid	C_7_H_6_O_5_	1.51	HR-MS/MS	169.0142
2	Protocatechuic acid	C_7_H_6_O_4_	2.88	HR-MS/MS	153.0193
3	Vanillic acid	C_8_H_8_O_4_	6.70	HR-MS/MS	167.0350
4	*p*-Coumaric acid	C_9_H_8_O_3_	9.72	HR-MS/MS	163.0401
5	Umbelliferone	C_9_H_6_O_3_	9.98	HR-MS	161.0244
6	Ferulic acid	C_10_H_10_O_4_	11.55	HR-MS/MS	193.0506
7	Apigenin 7-glucoside	C_21_H_20_O_10_	17.42	HR-MS/MS	431.0984
8	Kaempferol-3-O-glucoside	C_21_H_20_O_11_	17.89	HR-MS/MS	447.0933
9	Luteolin	C_15_H_10_O_6_	21.48	HR-MS	285.0405
10	Apigenin	C_15_H_10_O_5_	23.69	HR-MS	269.0455
11	Acacetin	C_16_H_12_O_5_	28.70	HR-MS/MS	283.0612

* Charge state is −1 for all ions.

**Table 2:** Targeted LC-HRMS(/MS) quantification of phenolic compounds confirmed with authentic reference standards–validation data.

No.	Compound Name	Calibration Slope	Calibration Intercept	Correlation Coefficient (r)	LOD (ug/ml)	Intra Day Precision at LOQ, % (*n* = 3)	Inter Day Precision at LOQ, % (*n* = 3)	Accuracy at LOQ, % (*n* = 3)
1	Gallic acid	99,709	−728	0.9954	0.010	6.34	6.80	109.1
2	Protocatechuic acid	82,174	3	0.9971	0.007	4.07	9.24	101.8
3	Vanillic acid	41,160	77	0.9941	0.012	6.13	6.23	93.8
4	*p*-Coumaric acid	152,459	1173	0.9915	0.010	7.81	9.83	97.3
5	Umbelliferone	526,781	982	0.9938	0.008	6.87	10.54	98.3
6	Ferulic acid	66,113	542	0.9966	0.014	5.24	10.53	100.4
7	Apigenin 7-glucoside	193,361	−364	0.9959	0.008	7.16	10.70	90.7
8	Kaempferol-3-O-glucoside	119,900	−81	0.9928	0.010	4.66	10.51	108.5
9	Luteolin	204,249	1943	0.9968	0.009	7.02	6.00	88.2
10	Apigenin	212,381	511	0.9936	0.008	5.05	7.99	90.7
11	Acacetin	263,873	−738	0.9964	0.009	5.81	9.38	109.3

**Table 3:** Identification and quantification of polyphenolic compounds in JC-DEE by HPLC–MS.

Compound Class	Compound Name	Concentration (µg/mL)	Content in Dried JC-DEE (µg/g)
Hydroxybenzoic acids	Gallic acid	Detected, <LOQ	Detected, <LOQ
	Protocatechuic acid	1.070 ± 0.049	356.544 ± 16.401
	Vanillic acid	0.133 ± 0.011	44.246 ± 3.672
Hydroxycinnamic acids	*p*-Coumaric acid	0.073 ± 0.005	24.181 ± 1.523
	Ferulic acid	Detected, <LOQ	Detected, <LOQ
Coumarins	Umbelliferone	0.067 ± 0.003	22.437 ± 1.055
Flavonoid glycosides	Apigenin-7-glucoside	0.266 ± 0.023	88.781 ± 7.724
	Kaempferol-3-*O*-glucoside	Detected, <LOQ	Detected, <LOQ
Flavonoid aglycones	Luteolin	0.560 ± 0.046	186.746 ± 15.313
	Apigenin	1.971 ± 0.150	657.149 ± 49.943
	Acacetin	Detected, <LOQ	Detected, <LOQ

Concentrations are expressed as mean ± SD (*n* = 3).

**Table 4:** FTIR band positions and tentative functional-group assignments for JC-DEE.

Bond	Tentative Interpretation	Wavenumber [1/cm]
O–H stretch	Alcohols/phenols; possible contribution from water in the KBr pellet	3431.36
=C–H stretch	Alkenic and/or aromatic C–H	3078.39
C–H stretch	Aliphatic CH_2_/CH_3_ groups	2933.73
C=O stretch	Carbonyl-containing constituents; conjugation is plausible, but the carbonyl class is unresolved	1697.36
C=C stretch/H–O–H bend	Alkenic unsaturation; possible overlap with water bending in the pellet	1647.21
C=C stretch	aromatic skeletal C=C vibration (ring vibration)	1508.33
C–H deformation/ring vibration	Aliphatic CH_2_/CH_3_ bending; aromatic skeletal contribution possible	1471.69
C–H deformation/ring vibration	Aliphatic CH_2_/CH_3_ bending; aromatic contribution possible	1448.54
C–H deformation	CH_3_ symmetric deformation	1373.32
C–O stretch	Phenolic, ether, or ester C–O contributions cannot be distinguished here	1259.52
C–O stretch	Phenolic, ether, or ester C–O contributions cannot be distinguished here	1234.44
C–O/C–O–C stretch	Alcohol, ether, and/or ester groups; overlapping fingerprint region	1174.65
C–O stretch	Alcohol and/or ether groups; overlapping fingerprint region	1029.99
Out-of-plane C–H bend	Alkenic =C–H and/or aromatic C–H; substitution pattern not established	889.18
Out-of-plane C–H bend	Alkenic =C–H and/or aromatic C–H; substitution pattern not established	844.82
Out-of-plane C–H bend	Alkenic =C–H and/or aromatic C–H; substitution pattern not established	792.74
Low-frequency feature	Potential atmospheric CO_2_ contribution; not assigned to the fraction	661.58
Low-frequency feature	Weak fingerprint-region signal; unassigned	518.85

**Table 5:** Unvalidated exploratory DPPH spectrophotometric observations for JC-DEE and ascorbic acid, expressed as apparent absorbance reduction relative to the unmatched reference control.

Sample	Concentration [mg/mL]	Apparent Reduction at 5 s [%]	Apparent Reduction at 1200 s [%]
Ascorbic acid	0.0176	84.98 ± 0.01	86.74 ± 0.20
JC-DEE	0.1	27.25 ± 0.13	28.11 ± 0.11
	0.3	26.17 ± 0.05	29.37 ± 0.00
	0.5	27.28 ± 0.19	31.48 ± 0.04
	0.8	28.36 ± 0.33	33.63 ± 0.04
	1.0	28.36 ± 0.33	35.95 ± 0.00

Values are presented as mean ± SD from three within-day determinations. Sample concentrations refer to the solutions prepared before addition to the DPPH reagent; final sample concentrations in the reaction mixtures were tenfold lower. Apparent absorbance reduction was calculated relative to an unmatched DPPH reference control. Because the control was not volume- or solvent-matched and extract-specific blanks were unavailable, the reported percentages include unresolved dilution-related and potential sample-dependent optical contributions. Accordingly, these values represent unvalidated exploratory spectrophotometric indices. Values displayed with an SD of 0.00 reflect rounding to two decimal places.

**Table 6:** Inhibition-zone diameters obtained by disk diffusion for the *J. communis* diethyl ether fraction and positive controls against the tested microbial strains.

Microbial Strain	Inhibition Zone (mm) Achieved by the Tested Fraction	Inhibition Zone (mm) Achieved by the Positive Control	Inhibibition Zone (mm) Achieved by the Negative Control
*Staphylococcus aureus*	10	20 **	8 ****
*Streptococcus pyogenes*	10	20 *	8 ****
*Escherichia coli*	8	20 **	7 ****
*Pseudomonas aeruginosa*	8	19 **	6 ****
*Candida parapsilosis*	7	23 ***	6 ****

Positive controls: * gentamicin (120 µg/disk); ** gentamicin (10 µg/disk); *** fluconazole (25 µg/disk), **** ethanol–water mixture (1:1, *v*/*v*).

## Data Availability

The original contributions presented in this study are included in the article. Further inquiries can be directed to the corresponding authors.
